# The effects of Ginseng Java root extract on uterine contractility in nonpregnant rats

**DOI:** 10.14814/phy2.12230

**Published:** 2014-12-03

**Authors:** Catthareeya Sukwan, Susan Wray, Sajeera Kupittayanant

**Affiliations:** 1 Agricultural Program, Faculty of Science and Technology, Nakhon Ratchasima Rajabhat University, Nakhon Ratchasima, Thailand; 2 Department of Cellular and Molecular Physiology, Institute of Translational Medicine, University of Liverpool, Liverpool, U.K; 3 School of Physiology, Institute of Science, Suranaree University of Technology, Nakhon Ratchasima, Thailand

**Keywords:** Calcium, ion channels, myometrium, sarcoplasmic reticulum

## Abstract

Ginseng Java or *Talinum paniculatum* (Jacq.) *Geartn* has long been used in herbal recipes because of its various therapeutic properties. Ginseng Java is believed to be beneficial to the female reproductive system by inducing lactation and restoring uterine functions after the postpartum period. There are, however, no scientific data on verifying the effects on the uterus to support its therapeutic relevance. Therefore, the purpose of this study was to investigate the effects of Ginseng Java root extract and its possible mechanism(s) of action on uterine contractility. Female virgin rats were humanely killed by CO_2_ asphyxia and uteri removed. Isometric force was measured in strips of longitudinal myometrium. The effects of Ginseng Java root extract at its IC_50_ concentration (0.23 mg/mL) on spontaneous, oxytocin‐induced (10 nmol/L), and depolarized (KCl 40 mmol/L) contraction were investigated. After establishing regular phasic contractions, the application of Java root extract significantly inhibited spontaneous uterine contractility (*n *=**5). The extract also significantly inhibited the contraction induced by high KCl solution (*n *=**5) and oxytocin (*n *=**5). The extract also inhibited oxytocin‐induced contraction in the absence of external Ca entry (*n *=**7) and the tonic force induced by oxytocin in the presence of high KCl solution. Taken together, the data demonstrate a potent and consistent ability of extract from Ginseng Java root to reduce myometrial contractility. The tocolytic effects were demonstrated on both spontaneous and agonist‐induced contractions. The fact that force was inhibited in depolarized conditions suggests that the possible mechanisms may be blockade of Ca influx via L‐type Ca channels. The data in Ca‐free solutions suggest that the extract also reduces IP_3_‐induced Ca release from the internal store. These tocolytic effects do not support the use of ginseng to help with postpartum contractility, but instead suggest it may be helpful in reducing inappropriate uterine contractions, such as in threatened preterm delivery.

## Abstract (precis)

The data demonstrate a potent and consistent ability of extract from Ginseng Java root to reduce myometrial contractility. The tocolytic effects were demonstrated on both spontaneous and agonist‐induced contractions.

## Introduction

Preterm delivery is a significant risk to pregnant women worldwide. Approximately 28% of these premature babies die within the first week after birth (Lawn et al. 2006). Factors possibly contributing to but not completely explaining this unwanted outcome, involve a breakdown in the normal uterine quiescence with a short‐circuiting or overwhelming of the normal parturition cascade (Giles and Bisits 2007). Several medications are used clinically as uterine relaxant or “tocolytics”, including magnesium sulfate, indometacin, *β*_2_‐adrenergic receptor agonists, atosiban, progesterone, prostaglandin synthesis inhibitors, nitric oxide donors, and calcium (Ca) channel blockers (Wray 2007). These drugs are designed to restrain the contractions of uterus. However, there is still controversy about their effectiveness and long‐term safety, especially to fetus (Kim and Shim 2006). These considerations underlie the continued use of traditional medicinal plants or plant products. Along with the quest to obtain novel tocolytics from plants, one plant that has long been used in ancient folk medicine, particularly in the treatment of type‐2 diabetes, inflammatory skin problems, gastrointestinal disturbance, general body weakness, and reproductive disorders is Ginseng Java (*Talinum paniculatum* (Jacq.) *Geartn*) (Shimoda et al. 2001; Pak et al. 2005; Setyowati and Wardah 2011). Although, ginseng has been reported to influence the female reproductive system, its effects on uterine contractility have never been investigated to support its therapeutic values. Thus, this study was undertaken to investigate the effects of Ginseng Java root extract on uterine contraction and its possible mechanism(s) of action.

## Methods

### Plant materials

Ginseng Java samples were collected from where they grew under natural conditions in the northeastern area of Thailand during the month of November, 2010. The plants were botanically identified by the Royal Forest Department of Thailand and the voucher specimen (BKF174387) deposited for future references.

### Extraction and isolation

Roots were separated, cleaned, chopped into small pieces, and dried in a hot air oven. Diced pieces of plants were powdered and extracted with refluxing methanol in a Soxhlet apparatus for 12 h. The extract was filtrated, evaporated under a reduced pressure at low temperature in a rotary evaporator, and dried by a lyophilizer. The yield of root extract was 6.67%. The extract was then stored at −20°C until use.

The extract was subjected to qualitative phytochemical screenings to identify the various classes of phytochemical constituents using standard procedures as previously described (Tiwari et al. 2011). Using this method, it was confirmed that Ginseng Java root extract contained alkaloids, flavonoids, and phytosterols (Yulia and Razief 2005).

### Animal procedures

Animal cares and uses followed the guidelines of the Laboratory Animal Resources, National Research Council of Thailand. The experimental procedures were approved by the Institutional Animal Care and Use Committee, Suranaree University of Technology, Thailand.

### Isolated uterine preparation and tension measurement

Adult female virgin rats, weighing 200–250 g were humanely killed by CO_2_ asphyxia. Uteri were isolated and immediately placed in the physiological saline solution. Longitudinal myometrial strips of 10 mm × 1–2 mm ×0.5 mm were dissected. To measure force, a uterine strip was attached at each end to metal hooks, with one hook attached to a transducer under a resting tension of 1 g in an organ bath containing the physiological saline solution maintained at 37°C, pH 7.4, and aerated with 100% O_2_ (Smith et al. 2005).

The strips were allowed to equilibrate and commence spontaneous contractions. After a minimum 30‐min period of producing stable contractile activity, the extract or drugs were applied via the superfusate. The electrical signal from the transducer was amplified and converted to a digital signal and recorded on a computer using Chart software, as detailed elsewhere (Munglue et al. 2013).

### Chemicals and physiological solutions

The solvents and chemicals used were of analytical grade and obtained from Sigma^®^ (St. Louis, MO, USA) and Merck^®^ (Darmstadt, Germany). All stock solutions were prepared and stored in accordance with the guideline of the producer.

The physiological Krebs's solution with pH 7.4 was prepared with the following compositions (mmol/L): NaCl: 154.0; KCl: 5.4; CaCl_2_: 2.0; MgSO_4_: 1.2; glucose: 8.0; 4‐(2‐hydroxyethyl)‐1‐piperazineethanesulfonic acid (HEPES): 10.0. Ca‐free physiological Krebs' solution was prepared by omitting CaCl_2_, but adding 1 mmol/L ethylene glycol‐bis(2‐aminoethylether)‐N,N,N′,N′‐tetraacetic acid (EGTA) (Kupittayanant et al. 2002). High KCl (40 mmol/L) solution was made by iso‐osmotic replacement of NaCl (Noble and Wray 2002). Oxytocin was dissolved in distilled water and used at the final concentration of 10 nmol/L to produce a phasic contraction. Bay K8644, the L‐type Ca channel agonist; S‐(‒)‐1,4‐dihydro‐2,6‐dimethyl‐5‐nitro‐4‐[2‐(trifluoromethyl)‐phenyl]‐3pyridine carboxylic acid methyl ester, was dissolved in distilled water and used at the final concentration of 10 *μ*mol/L (Matthew et al. 2004; Wray 2007; Noble et al. 2009). Ginseng Java root extract was used by directly dissolving in the physiological saline solution.

### Experimental protocols

#### Dose dependency of the extract

Spontaneous uterine contractions were allowed to reach equilibrium in the bathing medium for at least 30 min. These rhythmic contractions were observed and used as the control values (100% contraction). The concentration‐response effects of the ginseng extract on spontaneous contractions were tested by increasing concentrations of the extract in a cumulative manner (0.1–0.5 mg/mL) for 30 min intervals. The median inhibition concentration (IC_50_ value; a concentration which produce 50% of the maximum inhibition of the area under the contraction; AUC) was calculated by using a nonlinear curves fitting program, Microcal Origin Software (Vergara‐Galicia et al. 2010), and the concentration of IC_50_ value was used throughout the study.

#### Effects of the extract on bay K8644‐induced contraction

To investigate whether the relaxant effects of the extract was dependent upon external Ca entry through L‐type Ca channels, Bay K8644 (the L‐type channel agonist) was used (Triggle and Janis 1987). Bay K8644 (1 *μ*mol/L) was applied for 30 min and then plant extract was added in the continued presence of Bay K8644 for 30 min and then the extract removed from the bathing solution and the recovery monitored.

#### Effects of the extract on high K‐induced contraction

To determine the effects of the extract on contractile response to high K‐induced contraction, the strips were stimulated by high K (40 mmol/L) for 40 min and this value was taken as the control (100% contraction). After this exposure, high K was washed off and spontaneous contractions resumed. To determine the extract effects, the strips were stimulated by high K again for 20 min; the extract was then added to the strips in the continued presence of high K for 20 min. At the end of the experiment, the bathing solution was replaced by the physiological saline solution and recovery monitored. In addition, in some experiments the protocol was reversed, the extract was applied to the contracting strips and then high K was added, in the continued presence of the extract.

#### Effects of the extract on oxytocin‐induced contraction

##### (a) In normal Ca Krebs's solution

To determine the contractile response of the uterus to oxytocin, after the equilibrium stage of spontaneous contractions, the uterine strips were superfusated with oxytocin (10 nmol/L) for 40 min and this value used as the control (100% contraction). After 40 min exposure, oxytocin was washed off and spontaneous contractions resumed. To determine the effects of the extract, the strips were stimulated by oxytocin for 20 min; the extract was then added to the strips in the continued presence of oxytocin. At the end of the experiment, the bathing solution was replaced by the physiological saline solution and the recovery monitored.

##### (b) In the present of high K

The contracting uterine strips were induced to the plateau stage by high K. The solution in the bath was further replaced by high K containing oxytocin and equilibrated for 30 min. After 30 min exposure, the bath was replaced by the saline solution and spontaneous contractions resumed. To determine the effects of the extract, after the maximum contraction response to high K and oxytocin was achieved, the extract was subsequently applied. At the end of the experiment, the bathing solution was replaced by the saline solution and the recovery monitored.

##### (c) In Ca‐free EGTA solution

To determine the effects of the extract on intracellular Ca release, Ca‐free solution (1 mmol/L EGTA) was used. After the equilibrium period of spontaneous contractions, the uterine strips were applied with Ca‐free solution for 10 min. In the continued presence of Ca‐free solution, oxytocin was added to the organ bath for 10 min. Ca‐free solution and oxytocin was replaced by the control saline solution and spontaneous contractions resumed. The uterine strips were applied again with Ca‐free solution along with the extract for 10 min. In the continued presence of Ca‐free solution plus the extract, oxytocin was added to the organ bath for 10 min.

### Statistical analysis

Contractility parameters examined were area under the contraction (AUC), amplitude, and frequency. The contractile parameters were measured for at least 20 min. Results were expressed as percentages of control contractions (i.e., the control is 100%). To test the tocolytic effects of Ginseng Java extract in the presence of agonists including Bay K8644, oxytocin, or high K, the contractile parameters were compared for 20 min between agonist alone (0–20 min) and agonist plus Ginseng Java extract (20–40 min). Throughout, data are presented as mean ± SEM and “*n*” represents the number of samples, each one from a different animal. The data were evaluated using Microcal Origin Software, and the differences between control and treatment periods were analyzed by paired Student's *t*‐test. Probability values of less than 0.05 (*P *<**0.05) were considered statistical significant.

## Results

### Concentration‐response effects of the extract on the parameters of spontaneous contractions

**Figure 1.**
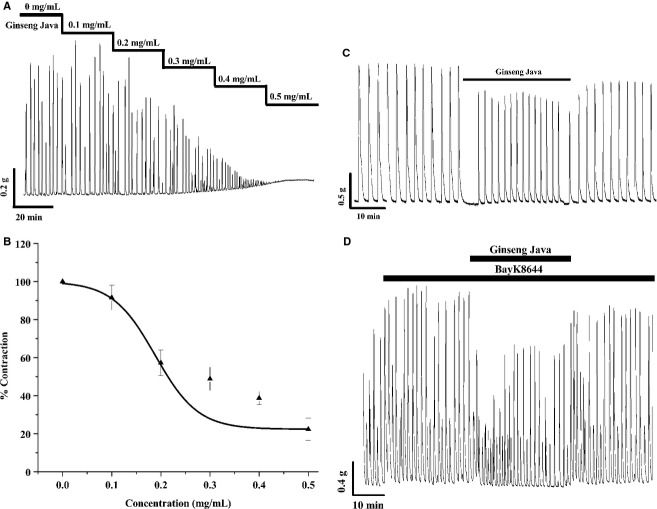
(A) The effects of increasing cumulative concentrations of Ginseng Java root extract (0–0.5 mg/mL) on spontaneous contractions of nonpregnant rat uterus. (B) A dose–response curve of Ginseng Java a root extract on uterine contractile activity. The IC_50_ of the extract was 0.23 mg/mL. Vertical bars represent the SEM (*n *=**5). (C) A trace represents the effects of Ginseng Java root extract on spontaneous contraction. (D) The trace representations of the effects of Ginseng Java root extract in the presence of the L‐type Ca channels activator (Bay K8644). The extracts show time‐dependent relaxations effect where Bay K8644 was added in a continued presence of the extract (*n *=**5).

Ginseng Java root extract inhibited spontaneous contractions in a concentration*‐*dependent manner (Fig. 1A). At each concentration (0.1–0.5 mg/mL), the amplitude of the spontaneous contractions was gradually reduced. With the highest dose, spontaneous contractions were essentially abolished, but a small and consistent tonic force was produced (*n *=**5). The IC_50_ for the amplitude of force was 0.35 mg/mL and for the area under the curve was 0.23 mg/mL (*R*^2^ 0.86) and Figure 1B shows the AUC mean data. This AUC IC_50_ value was used for subsequent experiments. As shown in Figure 1C, the relaxant effects of the Ginseng Java root extract at 0.23 mg/mL, were significant and maintained; spontaneous contracting isolated uterine strips decreased the amplitude, but slightly increased the frequency of contractions. The amplitude of contractions was significantly reduced to 60.6 ± 12.9% (*n *=**5), and the frequency of contractions was slightly but insignificantly increased to 109.2 ± 15.8% (*n *=**5), compared with 100% control. As a result of this, the AUC mean was, significantly decreased to 30.9 ± 4.5% compared with 100% control. It can be seen that the inhibitory effects of the extract were partially reversible over the time period examined, as upon return to physiological saline solution, the amplitude, frequency, and AUC were 84.6 ± 2.5%, 81.3 ± 3.6% and 76.5 ± 7% compared with 100% control. A typical trace of these experiments is shown in Figure 1C.

### Effects of ginseng java root extract in the presence of L‐type Ca channel agonist

Further experiments were designed to investigate whether Ginseng Java root extract could inhibit external Ca entry via L‐type Ca channels. To do so, external Ca entry via L‐type Ca channels was augmented by Bay K8644 (1 *μ*mol/L) and the contractile responses to the extract were observed (Fig. 1D). The results showed that as expected, Bay K8644 dramatically and significantly produced increases in the contractile responses compared with spontaneous contractions. Thus, the AUC, amplitude, and frequency in the presence of Bay K8644 was 125.3 ± 3.0%, 121.0 ± 3.4%, and 111.3 ± 3.2%, respectively, compared with 100% spontaneous control (*n *=**5). An addition of the extract in the continued presence of Bay K8644 produced tocolytic effects which significantly decreased the AUC and force amplitude (Fig. 1D). Thus, the AUC, amplitude, and frequency in the presence of Bay K8644 was 71.7 ± 14.6%, 75.0 ± 9.0%, and 119.1 ± 7.0%, respectively, compared with 100% Bay K8644 control (*n *=**5). As illustrated in Figure 1D, contractile activity reversed upon removal of the extract.

### Effects of ginseng java root extract on high K‐induced contraction

**Figure 2.**
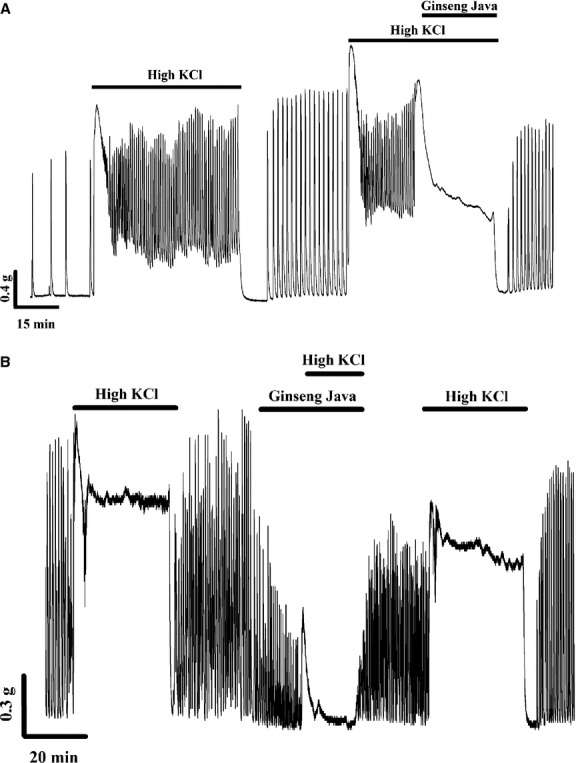
(A) The effects of Ginseng Java root extract on high KCl‐induced contraction. The extract was added after and in the continued presence of high KCl. (B) The effects of Ginseng Java root extract on high KCl‐induced contraction. The extract was added before and in the continued presence of high KCl.

We next investigated whether Ginseng Java root extract could affect force when the membrane was maintained depolarized using high K solution. As shown in Figure 2A, application of high KCl solution produced the expected rapid increase in force followed by a decline and rapidly oscillating force production (Crichton et al. 1993). An application of the ginseng extract dramatically altered this activity; it diminished force produced by high K, and stopped the oscillations. As shown in Figure 2A, 20 min after the extract application, the AUC had decreased to 76.1 ± 13.6% of control force development. When the extract was added before the application of high K, it could prevent the contraction in response to high K (see Fig. 2B). Thus, the AUC after the incubation of the extract and subsequently high K added was 27.7 ± 8.1%, compared with high K alone.

### Effects of ginseng java root extract on oxytocin‐induced uterine contraction

**Figure 3.**
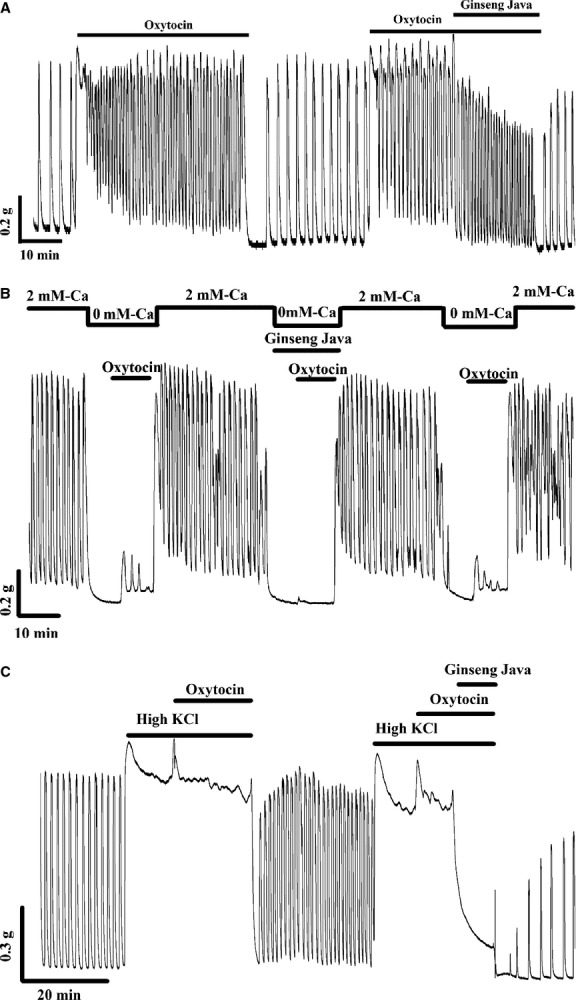
(A) The effects Ginseng Java root extract on oxytocin‐induced contraction. (B) The effects Ginseng Java root extract on oxytocin‐induced contraction in Ca‐free EGTA solution. (C) The effects Ginseng Java root extract on oxytocin‐induced contraction in the continued presence of high KCl.

The effects of Ginseng Java root extract on contraction induced by oxytocin (10 nmol/L) were investigated. As expected the contractile response of the uterine strips were augmented by oxytocin. Thus, the AUC, amplitude, and frequency in the presence of oxytocin were increased to 269.3 ± 13.0%, 109.8 ± 3.8%, and 128.4 ± 7.6%, compared with 100% spontaneous control (*n *=**5). Upon the extract application, a significant reduction in the AUC and amplitude of contraction occurred, (97.5 ±5.9%, 84.6 ± 4.9%, respectively) but not frequency compared with 100% oxytocin control. A typical trace is shown in Figure 3A.

The effects of the extract on the release of Ca from intracellular stores were also examined. In Ca‐free (EGTA) solution spontaneous contractions were abolished, as L‐type Ca channel entry of Ca is absent (Fig. 3B). Oxytocin produced a small amount of force, indicating the release of Ca from intracellular stores (Crichton et al. 1993). As can be seen in Figure 3B (typical of 6 other experiments), the extract prevented this contractile activity induced by oxytocin.

An application of oxytocin in the continued presence of high K produced a tonic contraction. Addition of the extract produced a noticeably drop in force. The AUC was approximately 72.2 ± 5.7% compared with those induced by oxytocin in the presence of high K (100%, *n *=**5). A typical trace of these experiments is shown in Figure 3C.

## Discussion

The outcome of this study demonstrated that Ginseng Java root extract has a strong tocolytic effect in rat myometrium. The extract could potently inhibit spontaneous, KCl‐ induced and oxytocin‐induced contraction. The extract could also inhibit force when only intracellular Ca sources were available, presumably the sarcoplasmic reticulum (SR). In addition, the extract could inhibit force under conditions of sustained, high Ca levels (depolarization and agonist). The effects of the extract could be partially reversed by administration of the activator of the L‐type Ca channels (see Fig. 1D). Thus, our conclusion is that the extract is a potent uterine relaxant acting via multiple mechanisms including inhibition of Ca entry through L‐type Ca channels and an inhibition of Ca release from the internal store.

Our phytochemical screening showed that Ginseng Java root extract contained alkaloids, flavonoids, and phytosterols which have also been found in previous studies (Yulia and Razief 2005; Thanamool et al. 2013). Numerous studies showed that these metabolites can relax activity in various types of smooth muscles. Zhang et al. (2012) demonstrated total alkaloids in *Buxus microphylla* leaf extract significantly relaxed thoracic aorta vascular smooth muscle, by suppressing influx of extracellular Ca via L‐type Ca channels and receptor‐operated Ca channels. Comparable to the findings of our study, Calixto et al. (1984) described how the alkaloids from *Phyllanthus sellowianus* extract, exhibited antispasmodic activity in rat uterus as well as in aortic ring and ileum smooth muscle. Additionally, the other alkaloids, for example, mitragynines, have also been reported to reduce KCl‐induced Ca influx in neuroblastoma cells (Matsumoto et al. 2005). Several effects of flavonoids on smooth muscle contraction have already been clearly described. Genistein and quercetin inhibited the vascular contractile activity induced by noradrenalin or serotonin (Di Salvo et al. 1993). They also reduced the spontaneous or agonist‐induced contractions in ileum smooth muscle (Herrera et al. 1992; Yang et al. 1992; Hollenberg 1994). Possible mechanisms included protein kinase inhibition (Srivastava 1985; Hollenberg 1993), increasing cAMP (Landolfi et al. 1984; Buxton 2004), inhibiting Ca influx (Di Salvo et al. 1993), and decreasing protein kinase activity (Duarte et al. 1994; Webb 2003). Plant phytosterols and their derivatives are known to affect the female reproductive system. These compounds can stimulate or inhibit uterine contraction based on the difference of their structures and which pathways they have most effect on, and how pure or crude the plant extracts under study are (Pulok et al. 2011). Phytosterols and saponins can act as inhibitors of the SR CaATPase and potassium channels, and in this way can increase the contractile activity (Bao et al. 2006; Promprom et al. 2010; Kupittayanant et al. 2014). Reports of uterine relaxant effects include Hsia et al. (2008) who demonstrated that fractionated phytosterols extracted from Adlay (*Coix lachryma‐jobi L. var. ma‐yuen Stapf*.) hull, attributed to block external Ca influx and Okunrobo et al.(2012) who reported that extracted saponins and alkaloids from *Pentaclethra macrophylla* produced significant inhibition of oxytocin in uterine smooth muscle. Taken together, our data indicated that the tocolytic effects of these plant extracts may be due to Ca antagonist activities of their phytosterols (Gilani et al. 1992; Revuelta et al. 1997). However, these interpretation and comparison are based on phytochemical screening. It is worth identifying the active compounds and studying their effects in the future.

It is well accepted that spontaneous contractions are dependent upon external Ca entry. Bay K8644 an L‐type Ca channel agonist can increase contraction by activating and opening L‐type Ca channels (Chien et al. 1966). Our studies showed that Ginseng Java root extract decreased contraction induced by Bay K8644 and the inhibitory effects were reversible (see Fig. 1D). This indicates that the extract inhibited the contraction via an inhibition of L‐type Ca channels.

Exposure of the uterine strips to high K solution provokes an increase in intracellular Ca concentration by depolarizing membrane potential, resulting in the opening of L‐type Ca channels, and hence contraction (Wray 2007). Some Ca channel antagonists can abolish the high K‐induced contraction (Grasa et al. 2004; Gharib Naseri and Yahyavi 2007; Lijuan et al. 2011). Applications of Ginseng Java root extract decreased force in the presence of high K solution (see Fig. 2A and B). Thus, our study suggests that Ginseng java root extract has the ability to block Ca entry when the channel is in the open state. The current model of Ca sensitization in smooth muscle contraction is accepted to be associated with G protein‐coupled receptor (GPCR) activation (Somlyo and Somlyo 2003; Shabir et al. 2004).

Various agonists including high K solution can produce contraction by coupling with GPCR, and relaxant agents can generate the opposite effect to cause Ca desensitization (Ratz et al. 2005; Ratz and Miner 2009). When the uterine strips were incubated with Ginseng Java root extract and subsequently high K solution, the strips could not produce force as much as induced by high K alone. Taken together, our finding also indicated that Ginseng Java root extract might cause Ca desensitization.

Oxytocin enhances uterine contractility by activating L‐type Ca channels and oxytocin receptor (Vrachnis et al. 2011). This receptor is connected to the GPCR which further activates phospholipase C and increases inositol 1,4,5‐triphosphate (IP_3_) production followed by promotion of Ca release from SR that leads to myometrial contraction (Sanborn et al. 1998). Our study demonstrated that Ginseng Java root extract significantly reduced contractions both in the presence of extracellular Ca source (see Fig. 3A) and when only the intracellular Ca source was available (see Fig. 3B). As the myometrium does not have functional ryanodine receptors (Dabertrand et al. 2006) and thus no Ca‐induced Ca release (Taggart and Wray 1998), this effect must be on IP_3_‐induced Ca release channels. Thus, our data clearly revealed that Ginseng Java root extract disrupted Ca entry and Ca release from SR via G‐protein signaling pathway. These two actions suggest the extract may act like an oxytocin receptor blocker in vivo.

Oxytocin‐induced contraction in the presence of high K solution is not only generated by Ca‐dependent pathways, but may also involve to a lesser extent Ca‐independent pathways via activation of Rho‐associated kinase (ROK) cascade (Somlyo and Somlyo 1998; Kupittayanant et al. 2001; Janssen et al. 2004). ROK mediated MLCP activity may be under the influence of oxytocin activity (Mitchell et al. 2013). However, the contraction triggered by ROK is more important in promoting tonic rather than phasic contractions (Ratz et al. 2005). In our studies, Ginseng Java root extract caused significant decreases in force during oxytocin‐induced contraction in the presence of high K (see Fig. 3C), suggesting the inhibitory effect of the extract involves the inhibition of ROK pathways. There may, however, be species differences in ROK and other pathways within smooth muscles and thus care must be taken in extrapolating this to human myometrium (Burdyga et al. 1995; Smith et al. 2002).

## Conclusion

Our studies on rat uterus provide the first evidence that Ginseng Java root extract produces tocolytic effects on both spontaneous and agonist‐induced contractions. The possible mechanism(s) may be due to the blockade of Ca influx via L‐type Ca channels, the inhibition of Ca release from the internal store, and alteration of the ROK pathway, that might reduce the sensitivity of contractile system to Ca. However, the effects of the extract should be specifically confirmed by investigating simultaneous measurements of force and Ca in uterine strips or electrophysiological methods in the uterine cells to see if the extract alters Ca changes or currents. Although our study is on nonpregnant rat myometrium, if these findings can be extrapolated to pregnant human myometrium the potent inhibitory effects of the extract on Bay K8644 and oxytocin‐induced contraction, could substantiate the medicinal use of Ginseng Java root to treat preterm labor or abnormal hyper‐contractility of the uterus. These tocolytic effects do not support the use of ginseng to help with postpartum contractility.

## Conflict of Interest

None declared.
